# Kaposiform hemangioendothelioma and tufted angioma: two entities of the same clinicopathological spectrum^☆^

**DOI:** 10.1016/j.abd.2021.07.010

**Published:** 2023-01-03

**Authors:** Lula María Nieto-Benito, Jorge Huerta-Aragonés, Verónica Parra-Blanco, Minia Campos-Domínguez

**Affiliations:** a Department of Dermatology, Hospital General Universitario Gregorio Marañón, Madrid, Spain; b Department of Pediatric Oncology and Hematology, Hospital General Universitario Gregorio Marañón, Madrid, Spain; c Department of Dermatopathology, Hospital General Universitario Gregorio Marañón, Madrid, Spain; d Laboratory of Immune-Regulation, “Gregorio Marañón” Health Research Institute (IISGM), Madrid, Spain; e Medical School, Universidad Complutense de Madrid, Madrid, Spain

Dear Editor,

kaposiform hemangioendothelioma (KHE) and tufted angioma (TA) are very rare vascular tumors^1^; however, they are associated with important morbidity and mortality.^2^ Their clinical presentation is very heterogeneous and, especially in KHE, potential associated complications add difficulties to the management.^1^, ^3^

A 28-day-old male infant, born at 33 weeks of gestation with a diagnosis of nonimmune *hydrops fetalis*, presented with an asymmetry of the right face, neck, and thorax after partial resolution of the generalized edema (Fig. 1A). On physical examination, an erythematous-bluish-purpuric vascular-like tumor extending from the right parotid and cervical area to the ipsilateral chest was observed. A diagnosis of KHE complicated with the kasabach-merritt phenomenon (KMP) was made through laboratory test results and magnetic resonance imaging (Fig. 1B). Intravenous treatment with vincristine, aspirin, ticlopidine and prednisone lead to the reduction in the size of the tumor and the improvement of the clinical condition.^3^ Aspirin and ticlopidine were maintained without any recurrence, symptomatic, or laboratory abnormalities. However, several months after discontinuation due to vaccination, dark-red and violaceous macules and plaques started to develop in the same location as prior HEK (Fig. 2).

**Figure 1:**
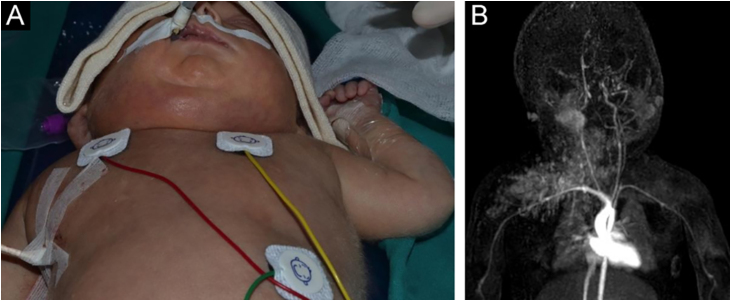
Clinical and radiological images of kaposiform hemangioendothelioma (KHE). (A) Clinical image at presentation: vascular-like lesion located at his right cervical area and chest. (B) Radiological image of a vascular tumor compatible with HEK with deep endocervical extension.

**Figure 2:**
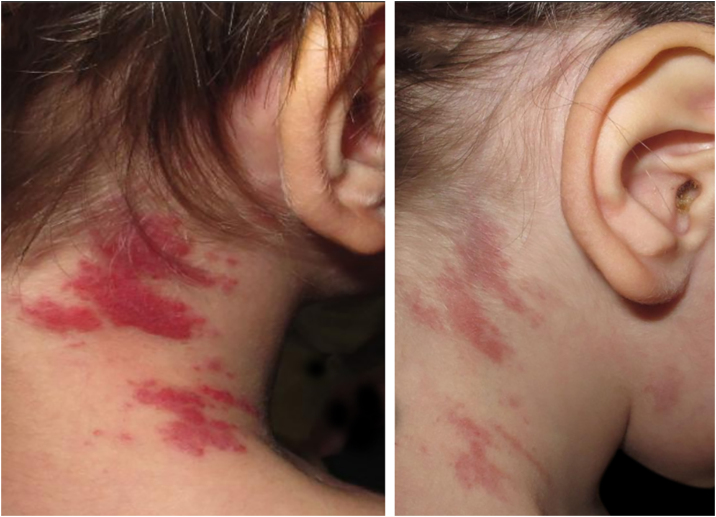
Clinical images of tufted angioma (TA) lesions. (A) Violaceous macules and plaques on the right lateral aspect of the neck at the age of two; first presentation of TA when discontinuation of aspirin and ticlopidine. (B) Partial clearance after reintroduction of aspirin in monotherapy.

Histopathological study from these lesions showed a vascular, well-defined nodular proliferation located in the papillary and medium dermis with a “cannonball” appearance. These nodules were formed by closely packed small vascular vessels lined with endothelial cells and pericytes; vascular spaces were present and some of them were located at the periphery, surrounding the nodules, with a semilunar/crescent-shaped morphology (Fig. 3). No mitotic figures, atypical cells or changes at the epidermis were present. An immunohistochemistry panel was also performed, and a diagnosis of TA was made. Aspirin was then reintroduced with clinical control and partial clearance (Fig. 2). New flares at the same location have been experienced when discontinuing aspirin, with complete control after reintroduction.

**Figure 3:**
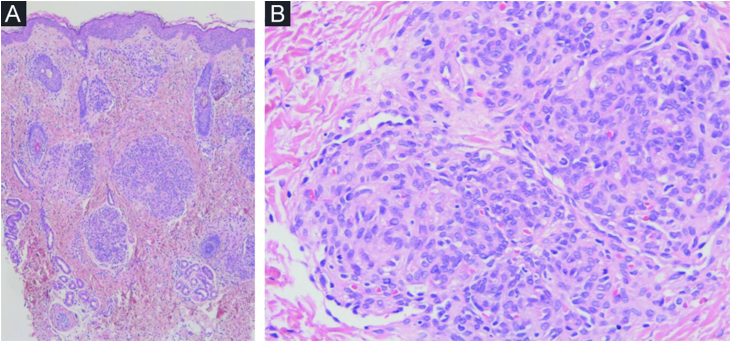
Histopathological features of tufted angioma (TA). (A) Vascular proliferation in the papillary and medium dermis; nodules were composed of tufted vascular vessels lined with endothelial cells. These endothelial cells were fVIII, CD31 and CD34 positive (podoplanin negative). (B) These nodules were surrounded at the periphery by semilunar vascular spaces (podoplanin positive).

HEK and TA are vascular tumors with aggressive and intermediate behavior, respectively.^1^, ^2^, ^3^ Regarding complications, KMP is the most severe. Both entities share clinical, histopathological, and molecular features (GNA14 mutations); therefore, it has been suggested to be two polar ends of the same spectrum.^2^ TA usually presents during infancy or early childhood with non-aggressive behavior.^2^, ^4^ Purpuric, dark red or brownish macules, papules and plaques are characteristic, although a deep nodular component or extension to the trunk and elbow can be observed.^1^, ^2^ The presence of dermal tufts of vessels in a cannonball pattern is pathognomonic (Fig. 3).^4^

Although recommended, histopathological studies can be omitted in life-threatening cases.^4^, ^5^ Concerning treatment, non-complicated, early and asymptomatic TA cases may not benefit from treatment.^1^, ^5^ Systemic corticosteroids, intravenous vincristine, mTOR inhibitors, α-interferon and antiplatelet drugs have been used successfully in the treatment of KHE/TA.^5^ VAT (Vincristin, Aspirin and Ticlopidine) therapy, as presented, has demonstrated its efficacy in cases of KHE/TA associated to KMP.^2^ Sirolimus, an mTOR inhibitor, has demonstrated great results in complicated, non-complicated, and refractory cases of KHE/TA.^1^, ^2^, ^5^

In conclusion, the authors present a case of neonatal KEH complicated with KMP, successfully treated with VAT therapy with posterior development of TA. TA relapses were experienced when discontinuing aspirin, with complete control after reintroduction in monotherapy.

## Financial support

None declared.

## Authors’ contributions

Lula María Nieto-Benito and Minia Campos-Domínguez conceived and designed the study, collected the data, and wrote and reviewed the paper. Jorge Huerta-Aragonés and Verónica Parra-Blanco conceived the study and reviewed the paper.

## Conflicts of interest

None declared.
